# The Effect of Intra-CA1 Agmatine Microinjection on Water Maze Learning and Memory in Rat

**Published:** 2011-05-01

**Authors:** K Rastegar, H Roosta, A Zarifkar, A Rafati, M Moosavi

**Affiliations:** 1 Shiraz Neuroscience Research Center and Department of Physiology, Shiraz University of Medical Sciences, Shiraz, Iran

**Keywords:** Agmatine, Water maze, Learning, Memory, Hippocampus, CA1, Rat

## Abstract

**Background:**

Reports on agmatine are controversial showing that it may improve memory, it can deteriorate memory and some did not notice any interference with learning and memory. In the present study, the effect of directly intra-CA1 agmatine microinjection on water maze learning and memory has been assessed.

**Methods:**

The cannuls were implanted in hippocampal CA1 regions of rats in a sterotaxic frame after general anesthesia. After one week recovery period, the animals were assessed in the reference memory version of water maze. Agmatine (1, 10, 100 or 200 μg/0.5 μl) or saline were infused 20 minutes before or immediately after training.

**Results:**

Agmatine-treated rats did not show any significant difference neither in water maze acquisition nor in consolidation task in comparison with control and sham groups.

**Conclusion:**

Agmatine does not affect water maze learning and memory.

## Introduction

Agmatine (4-aminobutyl guanidine) is one of the metabolites of L-arginine. It is synthesized by arginine decarboxylase (ADC) and hydrolyzed by agmatinase.[1] Although for a long time, it was considered as a constituent of bacteria, plants and invertebrates, later evidences suggested that agmatine exists also in mammalian tissues including brain.[2][3][4] It is widely distributed in the brain and its concentration is similar to the other neurotransmitters concentrations.[3][4][5] Because agmatine is synthesized in the brain, stored in synaptic vesicles, accumulated by uptake, released by depolarization and inactivated by agmatinase, it is considered to be a putative neurotransmitter.[6]

There are some evidences that agmatine modulate learning and memory. For example, agmatine (ip) has been reported to impair contextual fear conditioning and learning, but did not have any effect on water maze performance.[7][8] Arteni et al.[9] showed that agmatine (ip) facilitated animal's performance in inhibitory avoidance task. Intracerebroventricularly (icv) administered agmatine has been reported to modulate water maze reference memory dose-dependently; it improved memory at low dose (10 µg), but impaired it at a low dose (100 µg).[10] One of the brain structures which has an essential role in the processing of spatial learning is the hippocampus. The CA1 subregion as the last station of hippocampal neural loops, is specifically involved in spatial learning and memory.[11] Agmatine-like immunoreactivity has been detected in axon terminals of CA1 area, synapsing with pyramidal cells.[12] This agmatine is suggested to be co-stored with glutamate in hippocampal synaptic vesicles.[5] In hippocampal slices, agmatine superfusion decreases CA1 neural discharges.[13] In contrast, it is demonstrated that CA1 agmatine level increases after water maze spatial learning.[14]

Knowing the important role of CA1 region in hippocampal function, existence of agmatine in this area and the inconsistency of previous experiments regarding agmatine effect on hippocampal function, we aimed to target CA1 region to assess if agmatine can affect its function considering that intra-CA1 agmatine administration allows us to assess agmatine's direct effect on CA1 area with the least contribution of the other brain regions.

## Materials and Methods

Male Sprague-Dawely rats (200–250 g at the time of surgery) were obtained from Laboratory Animal Center of Shiraz University of Medical Sciences. The animals were housed four per cage before surgery and two per cage after surgery in a constant temperature (24±1°C) and humidity controlled room, under a 12-12h light/dark cycle. Food and water were provided ad libitum except for the periods of experimental studies in water maze. The behavioral experiments were done during the light phase. All experiments were carried out in accordance with recommendations from the Declaration of Helsinki and the internationally accepted principles for the use of experimental animals. Agmatine was purchased from Sigma (St. Louis, USA) and was diluted in sterile isotonic saline.

Animals were anesthetized with a combination of ketamine (100 mg/kg, ip) and xylazine (2.5 mg/kg, ip) and placed in a stereotaxic apparatus (Stoelting, USA). Stainless steel guide cannulae (22-gauge) were bilaterally implanted in the CA1 region with the following coordinates according to the rat brain atlas of Paxinos and Watson:[15] AP−3.84 mm from bregma, ML±2.2 mm from midline, DV−2.6 mm from the skull surface. The incisor bar was lowered 3.3 mm below horizontal zero to achieve the flat skull position. The cannulae were fixed to the skull surface with two stainless screws and dental acrylic cement. After surgery, all animals were allowed a 1-week post-operative recovery period before initiation of behavioral experiments.

Saline or drug were administered into CA1 region bilaterally, through guide cannula (22-gauge) using injection needles (27-gauge) connected by a piece of polyethylene tube to a 10 µl Hamilton syringe. The tip of the injection needle was protruded 0.5 mm beyond the guide cannula tip and in each microinjection 0.5 μl saline or drug was infused within 2 minutes. After completion of the infusion, the needle was left in place for another 60 seconds to allow diffusion of the drugs from the needle.

The water maze has been described previously.[16] Briefly it was a black circular pool with a diameter of 140 cm and a height of 70 cm, filled with 25±1°C water to a depth of 25 cm. The maze was divided geographically into four equal quadrants and release points that were designed at each quadrant as N, E, S, and W. A hidden circular platform (11 cm in diameter), was located in the center of the southwest quadrant and submerged 1.5 cm beneath the surface of the water. Fixed, extra maze visual cues were present at various locations around the maze (i.e. computer, a door, a window, bookshelves, posters). A CCD camera was mounted above the center of the maze so that the animal motion could be recorded and sent to the computer. The path of animal's swimming was automatically recorded by a computerized system (Noldus EthoVision, version 3.1) and then analyzed by computing several parameters, e.g. latency to find the platform, traveled distance as well as the swimming speed.

Experiment 1: The aim of experiment 1 was to determine the effect of bilateral pre-training injection of agmatine into CA1 region on water maze place learning. The rats were divided into 7 groups (n=7); four groups received bilateral injections of agmatine into CA1 area, 20 minutes before training in doses 1, 10, 100 and 200 µg/0.5 µl, the sham group received saline as vehicle (0.5 µl bilaterally) and the control group received nothing. In order to assess our water maze sensitivity, a positive control group receiving scopolamine in dose 1 mg/kg/ip- which previous studies had showed its memory impairing effect, was considered in the experiment. The time interval of 20 minutes was selected according to previous reports.[8]

One week after surgery and 20 minutes after saline or drug administration, the rats were trained in the water maze. Animals received four session training during four daily sessions;[17] each session consisted of four trials. During the first 3 days, the platform, situated in the center of the southwest quadrant, was submerged 1.5 cm below the surface of water and therefore invisible, for testing spatial learning. The platform position remained stable over 3 days and acquisition of this task was assessed. On day 4, the platform was elevated above water level, covered with a piece of aluminum foil and placed in the center of the southeast quadrant. This task was designed to assess animal’s motivation and sensorimotor coordination towards a visible platform. A trial was started by placing a rat in the pool and facing the wall of the tank. Each of four starting positions (north, east, south and west) was used once in a series of four trials and their orders were randomized. Each trial was terminated when the rat found and climbed onto the escape platform or when 60 s had elapsed. A rat was allowed to stay on the platform for 20 s; then the next trial was started. The animals which did not find the platform within 60 s, were put on the platform by the experimenter and were allowed to stay there for 20 s. After completion of the 4th trial, rats were gently dried with a towel and returned back to their home cage.

Experiment 2: The aim of experiment 2 was to determine the effect of bilateral post-training administration of agmatine into CA1 region on water maze memory consolidation. The rats were randomly divided into 7 groups (n=7); four groups received bilateral injections of agmatine into CA1 area, immediately after training in doses 1, 10, 100 and 200 µg/0.5 µl, the sham group received saline as vehicle (0.5 µl bilaterally) and the control group which received nothing. In order to assess our water maze sensitivity, a positive control group receiving scopolamine in dose 1 mg/kg/ip was put in the experiment.

One week after surgery, the rats were trained in the water maze. The single training session consisted of eight trials with four different starting positions that were equally distributed around the perimeter of maze.[16] The task requires rats to swim to the hidden platform guided by distal cues. After mounting the platform, the rats were allowed to remain there for 20 s and were then placed in a holding cage for 30 s until the start of the next trial. The animals were given a maximum of 60 s to find the platform; if they failed to find the platform in this time, they would be placed by the experimenter on the platform and allowed to stay there for 20 s. After completion of the training, the animals returned to their home cage until the retention testing (probe trial) 24 hours later. The probe trial consisted of a 60 s free swim period without a platform and the time spent in the target quadrant was recorded. Immediately after probe trial, the animals were given four trials for visuo-motor coordination on the visible platform.

At the completion of the memory test, animals were anesthetized with ether, decapitated and their brains were removed and stored in 10% formalin. After 2 days, the brain sections were prepared, mounted on slides, and cannulae placements were examined for verification of needle tip locations. Animals were included for data analysis only if both needle placements were located within the CA1 region.

Data were expressed as the mean±SEM. An analysis of variance (ANOVA) followed by Student, Newman-Keuls multiple comparison tests was used for statistical comparison. In all cases, a p value of less than 0.05 was considered statistically significant.

## Results

**Fig. 1A-C:**
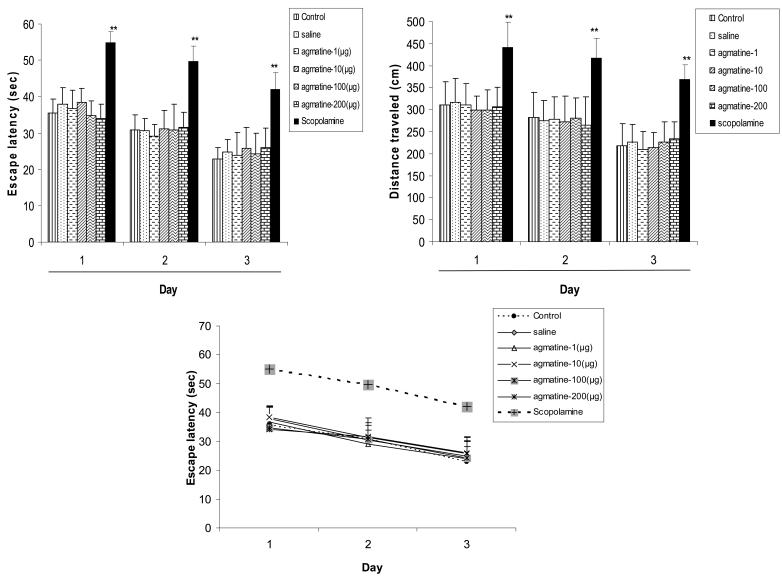
The effect of pre-training agmatine, saline or scopolamine administration on escape latency to the invisible platform during learning sessions (Fig. 1A); The effect of pre-training agmatine, saline or scopolamine administration on traveled distance to the invisible platform during learning sessions (Fig. 1B); and the learning patterns of the animals during learning sessions (Fig. 1C). Data are represented as mean±SEM. ** P<0.01 reveals significant difference between scopolamine treated and saline or control group.

Figure 1A shows the results obtained from daily pretraining infusion of saline or agmatine into CA1 area of the hippocampus on the escape latency to the hidden platform. Repeated measure ANOVA of the escape latency showed that pre-training intra-CA1 agmatine administration (1, 10, 100 and 200 μg) did not affect escape latency significantly in none of 3 days training in comparison to the respective days in saline treated and control group. However scopolaminetreated group significantly took longer time to reach the platform in comparison with saline and control group in those respective days.

Figure 1B shows the results obtained from pretraining infusion of saline or agmatine into CA1 area of the hippocampus on the traveled distance to the hidden platform. Repeated measure ANOVA of the escape latency showed that pre-training intra-CA1 agmatine administration (1, 10, 100 and 200 μg) did not affect traveled distance significantly in none of 3 days training in comparison to the respective days in saline treated and control group. However scopolamine increased this parameter in comparison with saline and control group in those respective days.

Figure 1C shows the learning pattern of the animals treated with saline or agmatine or scopolamine. This figure shows that there is a negative linear correlation between escape latency and the training sessions in all groups. This means that all groups have learnt the platform location; however scopolamine has slowed down learning capability.

**Fig. 2:**
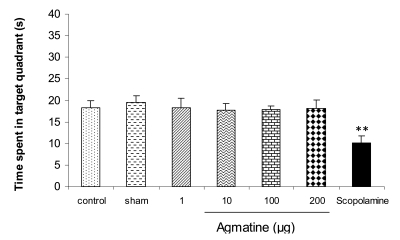
The effect of post-training agmatine, saline or scopolamine administration on the time traveled in target quadrant in probe test. The columns represent mean±SEM. ** P<0.01 reveals significant difference between scopolamine treated and saline or control group.

Figure 2 shows the results obtained from posttraining infusion of saline or agmatine into CA1 area of the hippocampus on memory consolidation in probe test. Post-training intra-CA1 agmatine administration (1, 10, 100 and 200 μg) did not affect the time animals spent in target quadrant in comparison to saline treated and control group although scopolamine treatment has decreased that. The data derived from single training session showed no difference between groups (data not shown).

The effect of pre- or post-training infusion of saline or agmatine into CA1 area of the hippocampus on the swimming speed of animals (data were averaged to obtain a mean performance of the groups) is depicted in Table 1. One way ANOVA of the mean animal’s swimming speed showed that intra- CA1 agmatine administration (1, 10, 100 and 200 μg) did not affect swimming speed significantly in comparison to saline treated group. Scopolamine treatment did not also affect the animal’s swimming speed in comparison to saline and control group. The effect of pre- or post-training infusion of saline or agmatine into CA1 area of the hippocampus on the escape latency to the visible platform is depicted in Table 2. One way ANOVA of the escape latency showed that intra-CA1 agmatine administration (1, 10, 100 and 200 μg) did not affect escape latency to the visible platform significantly in comparison to saline treated group. Also scopolamine treatment did not affect the escape latency to visible platform in comparison to saline and control group.

**Table 1:** The effect of pre-training and post-training agmatine, saline or scopolamine administration on animal’s swimming speed.

**Group**	**Control**	**Saline**	**Agmatine (μg)**				**Scopolamine**
			**1**	**10**	**100**	**200**	
pre-training							
Mean Speed (cm/s)	22.9	23.7	23.9	22.5	22.4	23.3	21.8
SEM	1	0.7	1.084	0.6064	1.595	1.2	1.77
post-training							
Mean Speed (cm/s)	25.5	25.8	26	24.75	24	26.6	24.2
SEM	1.9	1.68	1.65	1.03	1.27	1.25	1.3

**Table 2:** The effect of pre-training and post-training agmatine, saline or scopolamine administration on animal’s escape latency to the visible platform.

**Group**	**Control**	**Saline**	**Agmatine (μg)**				**Scopolamine**
			**1**	**10**	**100**	**200**	
pre-training							
Escape Latency (S)	14.5	15.75	16.7	16.3	17.1	15.8	16.3
SEM	3.1	2.9	1.85	3.3	1.9	2.95	1.4
post-training							
Escape Latency (S)	13.75	14.85	14.8	15.1	15.4	15.9	16.3
SEM	2.9	3.1	1.98	3.2	2.7	2.8	2.9

## Discussion

Our findings in the present study indicate that i) Pretraining intra-CA1 administration of 1, 10, 100 and 200 μg agmatine had no effect on water maze place learning, ii) Post-training intra-CA1 administration of agmatine in doses 1, 10, 100 and 200 μg could not affect water maze memory consolidation, iii) Intra- CA1 agmatine administration did not affect animal's swimming speed and iv) Intra- CA1 agmatine administration did not affect the escape latency to the visible platform in non-spatial visual discrimination task.

Agmatine did not affect animal's swimming speed or its motivation toward visible platform, showing that it does not affect animal's sensorimotor coordinance. Pre- or post-training administration of scopolamine (1 mg/kg/ip) could impair water maze learning and memory, which confirms our water maze system sensitivity. Agmatine was administered 20 minutes before each training episode, the time interval which previous studies reported some impairing effects after its intraperitoneal injection.[8] Pre-training agmatine administration was used to assess the effect of this drug on acquisition and possibly the consolidation process as the consolidation process of memory starts immediately after training till about 24 hours later. Becauses copolamine, as an impairing substance could deteriorate learning and memory in our essay, it seems reasonable to suggest that intra-CA1 agmatine microinjection does not affect water maze place learning. However to increase the accuracy of this study, in another experiment, agmatine was injected immediately after training to test its effect mainly on consolidation process. Although again post training scopolamine treatment impaired memory, post training agmatine administration had no effect on animal's memory.

Compatible with our results, McKay et al.[8] showed that agmatine (ip) did not affect water maze performance. In contrast, Liu et al.[10] reported that agmatine can affect water maze reference memory in a dose dependent manner; low dose agmatine (10 μg, icv) improved memory, while high dose agmatine (100 μg icv) produced impairment. The authors suggested that agmatine affects water maze reference memory in a dose dependent manner. Our study showed that agmatine administration to CA1 region of the hippocampus -in a wide range of doses- can not affect water maze reference memory. As CA1 is the last station of hippocampal synaptic loops, possibly agmatine has no effect on hippocampal function by itself. Interestingly, our recent study[17] showed that agmatine can protect hippocampus against LPSinduced spatial memory impairement and apoptosis. In that study periepheral agmatine administration (in doses 5 and 10 mg/kg) did not affect water maze performance, while it protected the animals against water maze placenavigation and hippocampal apoptosis. It was interesting for us to know if agmatine can affect hippocampal function in dose ranges wider than what we used in previous study. Then this study was designed to administer agmatine into CA1 area to know agmatine's direct effect on the hippocampus.

There are also some other experiments demonstrating the impairing effect of agmatine on learning and memory. For example it was shown that contextual fear conditioning and learning were impaired by agmatine (ip).[7][8] Although both contextual fear and water maze learning are dependent on the hippocampus, it is suggested that water maze learning requires the physiologic integrity of dorsal hippocampus,[18][19] whereas contextual fear learning appears to be based upon ventral hippocampal functioning.[19][20] Also, Otake et al.[4] who showed agmatine immunoblotting at numerous central sites have proposed that the highest agmatine concentration may be within subiculum (ventral hippocampus). As expected in our study, the cannulas were implanted in dorsal hippocampal CA1 regions.

Arteni et al.[9] found that agmatine (ip) facilitates memory consolidation in the inhibitory avoidance task. The systems and mechanisms responsible for passive avoidance task and water maze could be different. The structure which is more involved in inhibitory avoidance task is amygdale.[21] Amygdala receives its major excitatory inputs from locus coeruleus which is the principal source of agmatinecontaining cell bodies in the medulla oblongata.[4] Also Ruis-Duranetz et al.[22] demonstrated that agmatine icv infusion at doses of 10, 20 and 40 μg increased the firing rate of locus coeuleus neurons in a dose dependent manner.

In conclusion, our findings are the first report indicating that intra-CA1 agmatine administration in a broad range of doses has no effect on water maze learning and memory. It will be valuable in the future to know more about the effect of agmatine on the hippocampal function and mechanisms in physiologic and pathologic states as there are increasing evidences that agmatine is protective against some neurodegenerative disorders.
